# Supplementary material for: A comparison of dynamic balance performance between non-dancers and amateur dancers across three distinct dance genres: A cross-sectional study
Source: PLoS One. 2026 Feb 5;21(2):e0340621. doi: 10.1371/journal.pone.0340621 (PMC12875458; doi:10.1371/journal.pone.0340621)
Supplement: Attachment — Submitted filename: Response to Reviewer 2.docx [file pone.0340621.s003.docx]

**Response to Reviewer 2**

Thanks a lot for taking the time to review this manuscript and your valuable feedback. We have made changes and explaination as following. Please find the detailed responses below.

***Comments 1 While you have calculated p-values and Cohen’s d, some interpretations of significance vs. non-significance are inconsistent. For instance, some findings are labeled statistically significant despite a very small effect size (d < 0.2), which contradicts interpretation norms.***

**Response 1**

Thanks for your comments. We rewrited the sentence as follow:

“although the p value was smaller than 0.05 in DL, the Cohen’s d effect size was 0.09 which was smaller than 0.2, therefore demonstrating no significant main effect differences.” line 224-226

***Comments 2 Consider revising statistical interpretations with more caution regarding effect sizes.***

**Response 2**

Thank you for your valuable comments. In response, we have revisited and revised our interpretation of results where p-values were slightly above or near 0.05, but the corresponding effect sizes (Cohen’s d) exceeded 0.2. And we have utilized Cohen's d effect sizes (Cohen, 2013) to assess the magnitude of the differences, where small effects are defined as 0.20–0.49, moderate effects as 0.50–0.79, and large effects as >0.80. Additionally, differences were considered statistically significant at the p < 0.05 level, in line with standard practices in statistical analysis.

“although the p value was smaller than 0.05 in DL, the Cohen’s d effect size was 0.09 which was smaller than 0.2, therefore demonstrating no significant main effect differences.” line 224-226

***Comments 3 Although YBT is a strong test, it only measures reach and not center of mass control or reactive balance. Please acknowledge this as a limitation.***

**Response 3**

We thank the reviewer for this excellent and insightful point. We completely agree with this assessment. In direct response to this comment, we have added a new sentence to the 'Limitations' section of our manuscript to explicitly acknowledge this: “While this design choice strengthens the inference about recreational dance participation, it also means our findings cannot be directly compared to or explain the discrepant results often observed in studies of professional dancers. While the YBT is a widely accepted and reliable tool for assessing dynamic balance, it primarily measures reach distance rather than providing a direct evaluation of center of mass control or reactive balance responses. Future studies should consider complementing YBT with additional assessments, such as force plate analysis or reactive balance protocols, to provide a more comprehensive evaluation of balance performance.” line 378-382

***Comments 4 The Latin group had the lowest sample size (n=15), which may limit the power to detect differences. Please mention this limitation in the discussion section.***

**Response 4**

We added the sentence in Limitation section

“... , and the LAT group had the lowest sample size, which may limit the power to detect differences.”line 374-375

***Comments 5 The manuscript is generally understandable, but many sentences are overly long and would benefit from editing for clarity. A thorough language edit is strongly recommended to improve fluency and scientific tone.***

**Response 5**

We thank the reviewer for this valuable feedback. We agree that enhancing the clarity and fluency of the manuscript is important. In response, we have rewritten some sentence (even though it is for respond other comments, it helps to revised language edit as well) the introduction and discussion section in the manuscript, with marked in yellow.

***Comments 6 Some parts of the discussion are too descriptive and could benefit from more critical reflection. For instance, possible cultural, biomechanical, or training style reasons behind the non-significance in the anterior direction could be more deeply analyzed.***

**Response 6**

We sincerely thank the reviewer for their insightful comment and agree with their feedback.

We acknowledge certain limitations in the Discussion section. In the eighth paragraph, we provided a detailed comparison and analysis of the divergent results in the anterior direction and their potential causes. However, due to insufficient evidence, we were unable to establish a direct link between specific biomechanical characteristics and balance performance. In the interest of academic rigor, we therefore opted for a more objective description rather than engaging in further speculative interpretation. Additionally, we have revised the sixth paragraph of the Discussion and supplemented the Limitations section with corresponding clarifications.

“The results indicated that the scores of CHF and FLA were higher than those of LAT in the NDL for YBpm, but this difference did not appear in the dominant leg (DL). Although the reason for this result is not yet clear, some studies on dancer asymmetry might provide insights, and future research could explore this further. For example, Prus & Zaletel (2022) explored body asymmetries across four different dance genres: standard and Latin American dance, acrobatic rock and roll, breakdance, and hip-hop dance. The findings indicated that dancers in the standard and Latin American styles are the most prone to developing body asymmetries, primarily due to the closed positions characteristic of these dances and their inherent postural asymmetry (Prus & Zaletel, 2022). However, it is worth noting that this study grouped standard and Latin American dance together for analysis. While both styles belong to sport dancing and require partner coordination and feature asymmetrical closed positions, the authors suggest that differences may exist in movement patterns, physical demands, and training methods. Therefore, this aspect warrants consideration.” Line 309-320

**“Limitation and future perspectives**

While we observed significant associations between amateur dance participation and superior dynamic balance performance, we are unable to establish a causal relationship due to the cross-sectional nature of the study. To address this limitation and provide more robust evidence, future longitudinal studies tracking balance ability before and after individuals engage in dance training. Furthermore, this study focused exclusively on amateur dancers, and the LAT group had the lowest sample size, which may limit the power to detect differences. While this design choice strengthens the inference about recreational dance participation, it also means our findings cannot be directly compared to or explain the discrepant results often observed in studies of professional dancers. While the YBT is a widely accepted and reliable tool for assessing dynamic balance, it primarily measures reach distance rather than providing a direct evaluation of center of mass control or reactive balance responses. Future studies should consider complementing YBT with additional assessments, such as force plate analysis or reactive balance protocols, to provide a more comprehensive evaluation of balance performance.” Line 369-382

***Comments 7 Define abbreviations like YBcom, DL, NDL, etc., again in figure legends for clarity.***

**Response 7**

It was demonstrated in the previous version in line 214-218

***Comments 8 Ensure consistency in capitalization of terms like “control group” or “Latin group” across the manuscript.***

**Response 8**

As the reviewer noted, "Latin" in "Latin dance" is typically capitalized as it derives from a proper noun. Despite this, we agree that consistency in presentation is paramount for scientific rigor. We will ensure that the capitalization style for all group designations (e.g., control group, Latin dance group) is applied uniformly throughout the text, adhering to a single standard for readability and professionalism.

***Comments 9 Add a clearer rationale in the introduction for choosing those specific three dance genres.***

**Response 9**

Thanks for the comments, we added some sentence in the fifth paragraph in the Introduction section as following:

“However, research findings regarding the impact of dance on balance performance is not without controversy. Some evidences were found in Latin dance, flamenco dance and folk dance. The influence of Latin dance on balance performance remains a subject of debate that some studies demonstrated Latin dancers had better dynamic balance performance compared to non-dancers (Li et al., 2022; Liu et al., 2022), in contrast, Bojanowska et al. (2021) reported that the level of dynamic and static balances of Latin dancers was similar to non-dancers. For flamenco dance, one study separated fifty-two sedentary postmenopausal women randomly to a dance training group or self-care treatment advice group, and indicated a two-month intervention of flamenco and sevillanas training was effective in improving balance in sedentary postmenopausal women(Serrano-Guzmán et al., 2016), while Zhang et al. (2023) investigated flamenco dancers balance performance and reported that the score of the Y-Balance test (YBT) of amateur dancers was higher than professional dancers. Regarding folk dance, a study of professional folk dancers and non-dancers indicated that dancers had poorer dynamic balance than the non-dancers (Líška et al., 2024). Furthermore, a systematic review indicated when assessed through basic balance tests, dancers exhibited poorer balance compared to untrained individuals (Senger et al., 2024). For example, one study investigated balance for competitive dancers and control group for a single task (quiet stance) and a dual task (with a concurrent mental task) on a force plate, and indicated that the postural control of dancers and non-dancers appears to be similar (Kuczyński et al., 2011). ” line109-126
